# Asymptotic Identity in Min-Plus Algebra: A Report on CPNS

**DOI:** 10.1155/2012/154038

**Published:** 2011-06-29

**Authors:** Ming Li, Wei Zhao

**Affiliations:** ^1^School of Information Science & Technology, East China Normal University, No. 500 Dong-Chuan Road, Shanghai 200241, China; ^2^Department of Computer and Information Science, University of Macau, Avenue Padre Tomas Pereira, Taipa, Macau

## Abstract

Network calculus is a theory initiated primarily in computer communication networks, especially in the aspect of real-time communications, where min-plus algebra plays a role. Cyber-physical networking systems (CPNSs) are recently developing fast and models in data flows as well as systems in CPNS are, accordingly, greatly desired. Though min-plus algebra may be a promising tool to linearize any node in CPNS as can be seen from its applications to the Internet computing, there are tough problems remaining unsolved in this regard. The identity in min-plus algebra is one problem we shall address. We shall point out the confusions about the conventional identity in the min-plus algebra and present an analytical expression of the asymptotic identity that may not cause confusions.

## 1. Introduction

We use the term cyber-physical networking systems (CPNS) instead of cyber-physical systems (CPS) as that in Song et al. [1] for the meaning of Internet of Things (IoT) that was stated by Commission of the European Communities [2] or Networks of Things (NoT) as discussed by Ferscha et al. [3], intending to emphasize the point that we are interested in the networking theory in CPS. Communication networks in CPNS include, but are never limited to, the Internet. Physical systems considered in CPNS are heterogeneous, ranging from telemedicine systems to geophysical ones, see, for example, Clifton et al. [4], Traynor [5], Chang [6]. Obviously, data in various physical systems are heterogeneous, see, for example, Chang [6], Goodchild [7], Lai and Xing [8], Mandelbrot [9–11], Hainaut and Devolder [12], Cattani [13–17], Chen et al. [18–22], Mikhael and Yang [23], Bakhoum and Toma [24–26], Li [27–32], Li et al. [33–39], Messina et al. [40], Humi [41], Dong [42], Liu [43], Toma [44], Abuzeid et al. [45], [46–49], Werner [50], and West [51], just naming a few. 

There are two challenge issues in CPNS. On the one hand, data models that are irrelevant of statistics of a random function *x*(*t*) are greatly desired. On the other hand, theory that may be used to linearize nonlinear data transmission systems but irrelevant of their nonlinearity is particularly expected, because communication systems, including the Internet, are, in nature, nonlinear due to queuing, see, for example, Akimaru and Kawashima [52], Yue et al. [53], Gibson [54], Cooper [55], Pitts and Schormans [56], McDysan [57], and Stalling [58]. In short, we are interested in *data models that are irrelevant of their statistics and system theory that is irrelevant of the nonlinearity of systems*. 

The early work regarding the above in italic may refer to Cruz [59–61], Zhao and Ramamritham [62], Raha et al. [63], Chang [64, 65], Boudec [66], Boudec and Patrick [67], Firoiu et al. [68], and Agrawal et al. [69]. Following Cruz [59, 60], the theory for the above in italic is called network calculus, see, for example, [66, 67], Jiang and Liu [70]. Chang [71] uses the term (*σ*, *ρ*) calculus, which is taken as the synonym of network calculus of Cruz in this paper.

The main application area of network calculus is conventionally to computer science, the Internet in particular, see, for example, Wang et al. [72, 73], Li and Zhao [74, 75], Fidler [76], Jiang [77], Jiang et al. [78], Liu et al. [79], Li et al. [80], Li and Kinghtly [81], Burchard et al. [82], Ng et al. [83], Raha et al. [84, 85], Starobinski and Sidi [86], Fukś et al. [87], Jia et al. [88], Golestani [89], and Lenzini et al. [90]. However, we have to emphasize the point that its applications are never limited to computer science. Rather, it is a theory to model data irrelevant of their statistics and to deal with data transmission without the necessity in principle to consider the nonlinearity of transmission systems, as we shall explain in the next section. Therefore, it may be a promising tool to deal with data and systems in CPNS.

Basically, the fundamental theory of network calculus consists of three parts as described below.

- (*σ*, *ρ*) model of arrival data *x*(*t*),
- relationship between *x*(*t*), single system (or node or server) *S*(*t*) that is usually called service curve, and departure data *y*(*t*),
- departure data *y*(*t*) of a series of systems (nodes or servers) *S*_*n*_(*t*)  (*n* = 1,2,…), driven by arrival data *x*(*t*),

where min-plus algebra plays a role, see, for example, [66, 67, 70, 71, 76].

The contributions of this paper are in the following three aspects:

- the problem statement,
- the proof of the existence of the identity in the min-plus algebra in the domain of generalized functions,
- the asymptotic expression of the identity.

The rest of paper is organized as follows. Research background is discussed in Section 2. In Section 3, we will brief the min-plus algebra and state the problem regarding the identity in this algebra system. In Section 4, we shall address the existence of the identity in the min-plus algebra. The asymptotic expression of the identity is presented in Section 5. Discussions are given in Section 6, which is followed by conclusions.

## 2. Research Background

Data in CPNS are heterogeneous. They may be from sensors like radio-frequency identification (RFID), see, for example, [91], Ilie-Zudor et al. [92], Ahuja and Potti [93], data traffic in the Internet [38], transportation traffic (see [94–98]), ocean waves (see [31]), sea level (see [36, 99]), medical signals (see [14]), hydrological data (see [100]), financial data (see [101]), and so on. They may be Gaussian (see [29, 31]) or non-Gaussian (see [12, 102]). They may be in fractional order or integer order. In the case of fractional order, they may be unifractal or multifractal. The sample size of data of interest may be long enough for statistical analysis or very short, for example, a short conversation in mobile phone networks. On the other side, systems are also heterogeneous. Therefore, CPNS challenges us two tough issues. One is in data modeling and the other system modeling. We shall exhibit that the min-plus algebra in network calculus may yet serve as a tool in this regard. 

### 2.1. Network Model

We first explain a single node in CPNS. Then, a model of tandem network is mentioned. 

#### 2.1.1. Nonlinearity of Node in CPNS

**Figure 1:**
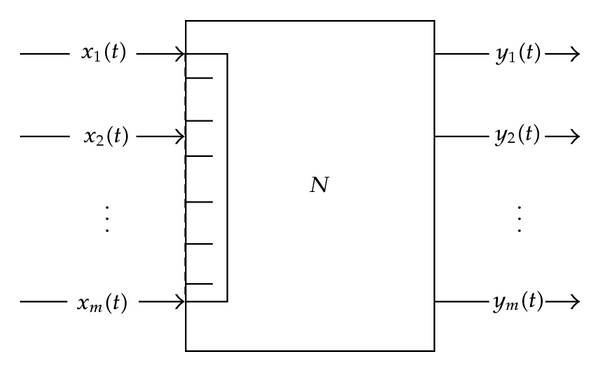
Single node in CPNS.

Denote by *N* a node in CPNS, see Figure 1. Suppose there are *m* clients arriving at the input of *N* at time *t*, see, for example, Starobinski et al. [103].

Without confusions, we use *N* to represent the operator of node *N* such that

(1) $$y_{i}\left(t\right)=\;Nx_{i}\left(t\right),\;1\leq i\leq m.$$

Recall that queuing is a phenomenon often occurring in CPNS. For instance, cars in highways are often queued. Clients in a library for borrowing or returning books need queuing. Suppose client *x*_*i*_(*t*) suffers from delay *d*_*i*_(*t*). Then,

(2) $$y_{i}\left(t\right)=x_{i}\left(t+d_{i}\left(t\right)\right),\;1\leq i\leq m.$$

Note that *d*_*i*_(*t*) is a random variable in two senses. One is

(3) $$d_{i}\left(t\right)\neq d_{j}\left(t\right),\;1\leq i\leq m,\;\;1\leq j\leq m,\;\;i\neq j.$$

The other is

(4) $$d_{i}\left(t_{1}\right)\neq d_{i}\left(t_{2}\right),\;1\leq i\leq m,\;\;t_{1}\neq t_{2}.$$

Therefore, we have the following remark.

**Remark 1 (nonlinearity)**

A node *N* in CPNS is usually nonlinear. That is,

(5) $$\sum y_{i}\left(t\right)\neq \sum Nx_{i}\left(t\right),\;1\leq i\leq m.$$

#### 2.1.2. Number of Arrivals is Random

The number of arrivals, denoted by *m* in Figure 1, is random.

**Note 1**

We need theory to deal with a nonlinear node *N* with *m* arrival clients, where *m* is a random variable.

#### 2.1.3. Tandem Network Model

**Figure 2:**
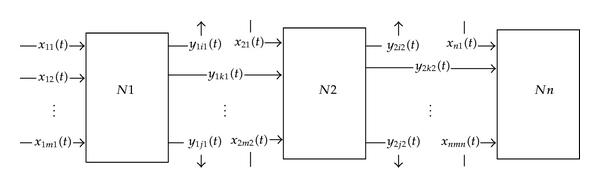
Tandem network.

A single node previously described is not enough in CPNS since a client may be served by a series of *n* nodes, which we call tandem network, see Figure 2.

According to Remark 1, each node in Figure 2 is nonlinear. In addition, considering Note 1, we see that the number of arrival clients at the input of each node is random. Some clients may go through from *N*1 to *Nn* while others may not. For instance, client *x*_1*i*1_(*t*) leaves the tandem network when it passes through *N*1. Further more, some clients, for example, *x*_21_(*t*), arrive at this tandem network at the input of *N*2. In general, how many clients leave the tandem network at the output of a specific node and how many clients arrive at the input of another specific node are uncertain.

**Note 2**

We need theory to handle a nonlinear system that is a tandem network as that in Figure 2 to assure the quality of service (QoS) of a specific client or of a specific class of clients within a given period of time.

The above Note 1 and Note 2 propose two challenge tasks in system theory. We shall explain how min-plus algebra is capable of dealing with those tasks late.

### 2.2. Data Modeling

We consider two classes of data flow. One is arrival data in the aggregated case, or aggregated clients, and the other arrival data of a specific client. In terms of network communications, the former is usually called aggregated arrival traffic while later arrival traffic at connection level. Without confusions, we use the term traffic rather than client. 

One of radical properties of arrival traffic (traffic for short) is remarked below.

**Remark 2 (positive)**

Traffic *x*_*i*_(*t*) is positive. That is,

(6) $$x_{i}\left(t\right)\geq 0,\;t\in R,$$

where **R** is the set of real numbers.

Another radical property of traffic is that the maximum of *x*_*i*_(*t*) is finite. More precisely, the value of *x*_*i*_(*t*) may never be infinite. Thus, we have the following remark.

**Remark 3 (finite range)**

The maximum of *x*_*i*_(*t*) is finite. That is,

(7) $$0\leq x_{i}\left(t\right)\leq x_{i,\max\;}.$$

**Remark 4 (randomness)**

The function *x*_*i*_(*t*) is usually random. This implies that

(8) $$x_{i}\left(t_{1}\right)\neq x_{i}\left(t_{2}\right)\;\text{for}\;t_{1}\neq t_{2}.$$

#### 2.2.1. Traffic at Connection Level

At connection level, for instance, for the *i*th connection, traffic is *x*_*i*_(*t*). One particularity of *x*_*i*_(*t*) is that *t* for *x*_*i*_(*t*) usually lasts within a finite time interval, say, [0, *T*]. The width of the interval may be short, such as a short conversation like a word “hello” or long, such as a long speech over a network. In any case, it is finite. Modeling *x*_*i*_(*t*) with short interval is particularly desired and challenging.

**Note 3**

In the discrete case, the length of *x*_*i*_(*t*) may be too short to the proper statistical analysis of *x*_*i*_(*t*) in practice.

**Note 4**

Without confusions, we use [0, *T*] to represent the interval in both the continuous case and the discrete one. In the continuous case, [0, *T*] ∈ **R**. In the discrete case, [0, *T*] ∈ **Z**, where **Z** is the set of integer numbers, implying *t* = 0,1,…, *T*. We use [*t*_1_, *t*_2_] to represent an interval the starting point of which is nonzero.

#### 2.2.2. Aggregated Traffic

We adopt Figure 1 to discuss aggregated traffic. At time *t*, aggregated traffic denoted by *x*(*t*) at a node is expressed by

(9) $$x\left(t\right)=\sum x_{i}\left(t\right),\;i=1,\ldots ,m.$$

In contrary to *x*_*i*_(*t*), the particularity of *x*(*t*) is that *t* for *x*(*t*) usually lasts within an interval longer than that of *x*_*i*_(*t*). As a matter of fact, if *x*_*i*_(*t*) passes through a node, another arrival flow *x*_*j*_(*t*)  (*j* = 1,…, *m*) may arrive at the node. Consequently, in general, we should consider *t* ∈ (0, *∞*) for *x*(*t*).

### 2.3. Accumulated Traffic

Traffic, either *x*_*i*_(*t*) or *x*(*t*), discussed previously is instantaneous one. Data modeling of instantaneous traffic is essential, as we need understanding what its behaviors are at instantaneous time *t* at the input of a node. However, from the point of view of the service of a node, we also need data modeling of accumulated traffic within a time interval, say, [0, *T*], without loss of generality, because it is desired for us to understand what the service performance of the node is for the purpose of proper design of a buffer size as well as scheduling policy of the node.

#### 2.3.1. Accumulated Traffic at Connection Level

In the continuous case, the accumulated traffic of *x*_*i*_(*t*) within the interval [0, *T*] is denoted by *X*_*i*_(*T*). It is given by

(10) $$X_{i}\left(T\right)=\overset{T}{\underset{0}{\int }}x_{i}\left(t\right)dt,\;t\in R.$$

In the discrete case,

(11) $$X_{i}\left(T\right)=\overset{T-1}{\underset{t=0}{\sum }}x_{i}\left(t\right),\;t\in Z.$$

#### 2.3.2. Accumulated Traffic in the Aggregated Case

Denote by *X*(*T*) the accumulated traffic in the aggregated case within the interval [0, *T*]. Then, in the continuous case, we have

(12) $$X\left(T\right)=\overset{T}{\underset{0}{\int }}x\left(t\right)dt,\;t\in R.$$

In the discrete case,

(13) $$X\left(T\right)=\overset{T-1}{\underset{t=0}{\sum }}x\left(t\right),\;t\in Z.$$

The mathematical expressions of *X*(*T*) and *X*_*i*_(*T*) appear similar except the subscript *i*. However, *X*(*T*) differs from *X*_*i*_(*T*) substantially in analysis in methodology. On the one hand, *T* for *X*_*i*_(*T*) should be assumed to be short such that conventional methods in statistics fail to its statistical analysis. On the other hand, *T* for *X*(*T*) may be large enough such that it may be sectioned for the statistical analysis, see, for example, Li et al. [104].

#### 2.3.3. A Basic Property of Accumulated Traffic

One property of accumulated traffic, either *X*(*T*) or *X*_*i*_(*T*), is the wide sense increasing. By wide sense increasing, we mean that

(14) $$X_{i}\left(T_{1}\right)\leq X_{i}\left(T_{2}\right)\;\text{for}\;T_{1}\leq T_{2},$$

or

(15) $$X\left(T_{1}\right)\leq X\left(T_{2}\right)\;\text{for}\;T_{1}\leq T_{2}.$$

Therefore, the data functions or series we face with are increasing ones in the wide sense.

#### 2.3.4. (*σ*, *ρ*) Model of Data

For *σ* ≥ 0 and *ρ* ≥ 0, the following is called the (*σ*, *ρ*) model of data *x*_*i*_(*t*),

(16) $$X_{i}\left(T\right)=\overset{T}{\underset{0}{\int }}x_{i}\left(u\right)du\leq \sigma_{i}+\rho_{i}T.$$

**Note 5**

The model expressed by (16) is irrelevant of any information of statistics of *x*_*i*_(*t*). The advantage of this model is at the cost of using inequality instead of equality.

**Note 6**

The model of (16) is simple in computation. Thus, it may be effective in practice, particularly in environments of CPNS, where simple computations are always expected.

For accumulated traffic *X*(*T*), we have

(17) $$X\left(T\right)=\overset{T}{\underset{0}{\int }}x\left(u\right)du\leq \sigma +\rho T.$$

Due to sufficiently large *T*, we may set the starting time by *T*_0_. In this case, we have

(18) $$\overset{T}{\underset{T_{0}}{\int }}x\left(u\right)du\leq \sigma \left(T_{0}\right)+\rho \left(T-T_{0}\right).$$

Moreover, we are allowed to section the above integral such that

(19) $$\overset{(n+1)T}{\underset{nT}{\int }}x\left(u\right)du\leq \sigma \left(nT\right)+\rho \left(T\right),\;n=0,1,\ldots .$$

Without loss of generality, we use (17) to explain *σ* and *ρ*.

**Remark 5**

The parameter *σ* represents the bound of the burstness or local irregularity of *x*(*t*), because

(20) $$0\leq \underset{T\to 0}{\lim\;}\overset{T}{\underset{0}{\int }}x\left(u\right)du\leq \sigma .$$

Note that the above integral does not make sense if lim _*T*→0_ ∫_0_^*T*^ *x*(*t*)*dt* ≠ 0 for the continuous *x*(*t*) even in the field of the Lebesgue's integrals, see Dudley [105], Bartle and Sherbert [106], and Trench [107] for the contents of the Lebesgue's integrals. However, it makes sense when it is considered in the domain of generalized functions, which we shall brief in the following section. A simple way to explain (20) is

(21) $$\underset{T\to 0}{\lim\;}\overset{T}{\underset{0}{\int }}x\left(t\right)dt=\overset{T}{\underset{0}{\int }}\sigma_{1}\delta \left(t\right)dt,$$

where *σ*_1_ ≤ *σ* and *δ*(*t*) is the Dirac-*δ* function.

**Remark 6**

The parameter *ρ* represents the bound of the average rate of *X*(*T*), because

(22) $$0\leq \underset{T\to \infty }{\lim\;}\frac{\int_{0}^{T}x\left(t\right)dt}{T}\leq \rho =\text{constant}.$$

**Remark 7**

The parameter *σ* measures the local property of *x*(*t*) while *ρ* is a measure of global property of *x*(*t*).

## 3. Min-Plus Algebra and Problem Statement

Min-plus convolution is essential in the min-plus algebra. In this section, we first briefly review the conventional convolution in linear systems. Then, we shall visit min-plus convolution. Finally, we shall state the problem in the aspect of identity in the min-plus algebra.

### 3.1. Conventional Convolution

Denote by *p* a real number that satisfies 1 ≤ *p* < *∞*. If a function *f*(*t*) defined on [*a*, *b*], where *a* is allowed to be −*∞*  and *b* is allowed to be *∞*, is measurable and

(23) $$\overset{b}{\underset{a}{\int }}{\left|f(u)\right|}^{p}du<\infty ,$$

we say that *f*(*t*) ∈ *L* ^*p*^(*a*, *b*).

Suppose that two functions *f*_1_(*t*), *f*_2_(*t*) ∈ *L* ^1^(−*∞*, *∞*). Then, one says that *f*_1_(*t*) convolutes *f*_2_(*t*) if

(24) $$f_{1}\left(t\right)*f_{2}\left(t\right)=\overset{\infty }{\underset{-\infty }{\int }}f_{1}\left(u\right)f_{2}\left(t-u\right)du,$$

where ∗ is the symbol implying the operation of convolution. We call it conventional convolution so as to distinguish it from the min-plus convolution we are discussing in this paper.

The conventional convolution is crucial for linear systems, see, for example, Gibson [54], Box et al. [108], Mitra and Kaiser [109], Papoulis [110], Harris [111], Mikusinski [112], Fuller [113], and Bendat and Piersol [114], just naming a few. It has the properties described by the following lemmas.

**Lemma 1**

In the algebra system (*L* ^1^; ∗), the conventional convolution is commutative.

**Lemma 2 (closure of ∗)**

If *f*_1_(*t*), *f*_2_(*t*) ∈ *L* ^1^, then *f*_1_(*t*)∗*f*_2_(*t*) ∈ *L* ^1^.

**Lemma 3**

In the algebra system (*L* ^1^; +, ∗), where + implies the ordinary addition, ∗ with respect to + is distributive.

**Lemma 4**

For *a* ∈ **R**, [*af*_1_(*t*)]∗*f*_2_(*t*) = *f*_1_(*t*)∗[*af*_2_(*t*)] = *a*[*f*_1_(*t*)∗*f*_2_(*t*)].

**Lemma 5**

The identity in (*L* ^1^; ∗) is the Dirac-*δ* function *δ*(*t*) that is defined by

(25) $$f\left(t\right)=\overset{\infty }{\underset{-\infty }{\int }}f\left(u\right)\delta \left(t-u\right)du,$$

where *f*(*t*) ∈ *L* ^1^(−*∞*, *∞*) is continuous at *t*.

In fact, in the domain of generalized functions, we have

(26) $$\overset{\infty }{\underset{-\infty }{\int }}\delta \left(u\right)du<\infty .$$

Thus, *δ*(*t*) ∈ *L* ^1^(−*∞*, *∞*)  in the sense of generalize functions. Consequently, *δ*(*t*) is taken as the asymptotic identity in (*L* ^1^; ∗) in the domain of generalized functions. Accordingly, the inverse of the conventional convolution discussed by, for instance, Mikusinski [112], Bracewell [115], Huang and Qiu [116], Abutaleb et al. [117], Rhoads and Ekstrom [118], Todoeschuck and Jensen [119], and Moreau et al. [120], exists because the necessary and sufficient condition that the inverse of an operation exists is that there exists the identity in that system, see, for example, Korn and Korn [121], Zhang [122], Riley et al. [123], Bronshtein et al. [124], and Stillwell [125], but it should be in the sense of generalized functions. As a matter of fact, the conventional convolution itself is in that sense, see, for example, Smith [126].

**Theorem 1**

The algebra system (*L* ^1^; ∗) is a group.

**Proof**

First, the operation ∗ is closed in *L* ^1^. Second, ∗ is commutative because, for any *f*_1_(*t*), *f*_2_(*t*), *f*_3_(*t*) ∈ *L* ^1^(−*∞*, *∞*),

(27) $$f_{1}\left(t\right)*\left[f_{2}\left(t\right)*f_{3}\left(t\right)\right]=\left[f_{1}\left(t\right)*f_{2}\left(t\right)\right]*f_{3}\left(t\right).$$

Finally, there exists the left identity denoted by *δ*(*t*) and the right one again denoted by *δ*(*t*) in (*L* ^1^; ∗) such that

(28) $$f\left(t\right)*\delta \left(t\right)=\;\delta \left(t\right)*f\left(t\right)\;\text{for}\;\text{any}\;f\left(t\right)\in L^{1}\left(-\infty ,\infty \right).$$

Thus, (*L* ^1^; ∗) is a group.

### 3.2. Min-Plus Convolution

Considering the property of wide sense increasing of accumulated traffic mentioned in Section 2.3, we denote by *𝕊* the set that contains all functions that are greater than or equal to zero and that are wide sense increasing.

**Definition 1**

Let *X*_1_(*t*), *X*_2_(*t*) ∈ *𝕊*. Then, the following operation is called min-plus convolution:

(29) $$X_{1}\left(t\right)⊗X_{2}\left(t\right)=\underset{0\leq u\leq t}{\inf\;}\;\left\{X_{1}\left(u\right)+X_{2}\left(t-\;u\right)\right\},$$

where ⊗ represents the operation of the min-plus convolution.

**Example 1**

Let *X*(*t*) = *t* ^2^ for *t* > 0 and 0 elsewhere. Then, *X*(*t*) ⊗ *X*(*t*) = *t* ^2^/2.

**Lemma 6 . (closure of ⊗)**

Let *X*_1_(*t*), *X*_2_(*t*) ∈ *𝕊*. Then, *X*_1_(*t*) ⊗ *X*_2_(*t*) ∈ *𝕊*.

**Lemma 7**

The operation ⊗ is commutative. That is,

(30) $$X_{1}\left(t\right)⊗X_{2}\left(t\right)=X_{2}\left(t\right)⊗X_{1}\left(t\right)\;\text{for}\;X_{1}\left(t\right),X_{2}\left(t\right)\in 𝕊.$$

Define another operation that is denoted by ∧ such that

(31) $$X_{1}\left(t\right)\wedge X_{2}\left(t\right)=\inf\;\left[X_{1}\left(t\right),X_{2}\left(t\right)\right]\;\text{for}\;X_{1}\left(t\right),X_{2}\left(t\right)\in 𝕊.$$

Then, we have an algebra system denoted by (*𝕊*, ∧, ⊗) that follows the distributive law.

**Lemma 8**

The operation ⊗ with respect to ∧ is distributive. That is, for *X*_1_(*t*), *X*_2_(*t*), *X*_3_(*t*) ∈ *𝕊*, one has

(32) $$\left[X_{1}\left(t\right)\wedge X_{2}\left(t\right)\right]⊗X_{3}\left(t\right)=\left[X_{1}\left(t\right)⊗X_{3}\left(t\right)\right]\wedge \left[X_{2}\left(t\right)⊗X_{3}\left(t\right)\right].$$

The following rule useful in this research is stated as follows.

**Lemma 9**

Suppose *K* ∈ **R**. Then, for *X*_1_(*t*), *X*_2_(*t*) ∈ *𝕊*, one has

(33) $$\left[X_{1}\left(t\right)+K\right]⊗X_{2}\left(t\right)=X_{1}\left(t\right)⊗X_{2}\left(t\right)+K,$$

where + is the ordinary addition.

Denote by *I*_1_(*t*) the conventional identity in the min-plus algebra, which is defined by

(34) $$I_{1}\left(t\right)=\{\begin{array}{cc} \infty , & t>0,\\ 0, & t<0, \end{array}$$

see [66–70]. 

It seems quite obvious when one takes *I*_1_(*t*) as the identity in the min-plus algebra since

(35) $$X\left(t\right)⊗I_{1}\left(t\right)=\;I_{1}\left(t\right)⊗X\left(t\right)=X\left(t\right).$$

However, we shall soon point the contradictions of *I*_1_(*t*) below.

### 3.3. Problem Statement

Denote by *u*(*t*) the Heavyside unit step function. That is,

(36) $$u\left(t\right)=\{\begin{array}{cc} 1, & t>0,\\ 0, & t<0. \end{array}$$

Then, for *K* ∈ **R**, we have

(37) $$Ku\left(t\right)=\{\begin{array}{cc} K, & t>0,\\ 0, & t<0. \end{array}$$

Using (34), we have

(38) $$\begin{array}{cc} I_{1}\left(t\right)+Ku\left(t\right) & =\{\begin{array}{cc} \infty +K, & t>0\\ 0, & t<0 \end{array}\\ & =\{\begin{array}{cc} \infty , & t>0\\ 0, & t<0 \end{array}\\ & =I_{1}\left(t\right). \end{array}\;\left(\text{Contradiction}\;1\right)\;$$

The above is an obvious contradiction regarding the conventional identity defined by (34). 

In addition to the above contradiction, we now state another problem regarding (34). As a matter of fact, if we let *X*_1_(*t*) = *I*_1_(*t*) and *Ku*(*t*) in Lemma 9, then, on the left side of (33) in Lemma 9, we have

(39) $$\left[I_{1}\left(t\right)+Ku\left(t\right)\right]⊗X_{2}\left(t\right)=I_{1}\left(t\right)⊗X_{2}\left(t\right)=X_{2}\left(t\right).$$

On the other side, on the right side of (33) in Lemma 9, we have

(40) $$\begin{array}{cc} \left[I_{1}\left(t\right)+Ku\left(t\right)\right]⊗X_{2}\left(t\right) & =I_{1}\left(t\right)⊗X_{2}\left(t\right)+Ku\left(t\right)\\ & =X_{2}\left(t\right)+Ku\left(t\right). \end{array}$$

Comparing the right sides of (39) with that of (40) yields another contradiction expressed by

(41) $$X_{2}\left(t\right)=X_{2}\left(t\right)+Ku\left(t\right),\;\left(\text{Contradiction}\;2\right)$$

The above discussions imply that the definition of the identity of (34) in the min-plus algebra, which is commonly used in literature, see, for example, [66–70], may not be rigorous at least. Therefore, the conventional representation of the identity, that is, (34), may be inappropriate since it may mislead computation results like those in (39) and (40). Consequently, rigorous definition of the identity needs studying.

## 4. Existence of Identity in Min-Plus Algebra

The problems regarding the definition of the conventional identity, which we stated in Section 3.3, give rise to a question whether or not the identity in the min-plus algebra exists. The answer to this question is rarely seen, to the best of our knowledge. Another question resulted from Section 3.3 is what the rigorous representation of the identity is. We shall provide the answer to the first question in this section. The answer to the second will be explained in the next section.

### 4.1. Preliminaries

We brief some results in generalized functions [127–129] for the purpose of discussing the existence of identity.

**Definition 2**

Let supp (*f*) be the support of a function *f* : **R** → **C**. It implies {*t* : *f*(*t*) ≠ 0}. The function is said to have a bounded support if there exist *a*, *b* ∈ **R** such that supp (*f*)⊂[*a*, *b*].

**Definition 3**

A function *f* : **R** → **C** is said to have *n* time continuous derivatives if its first *n* derivatives exist and are continuous. If its derivatives of all orders exist and are continuous, *f* is said to be infinitely differentiable. In this case, *f* is said to be smooth.

**Definition 4**

A test function is a smooth **R** → **C** with supp (*f*)⊂[*a*, *b*]. The set of all test functions is denoted by **D**.

**Definition 5**

A linear functional *f* on **D** is a map *f* : **D** → **C** such that, for *a*, *b* ∈ **C** and *ϕ*, *ψ* ∈ **D**, *f*(*aϕ* + *bψ*) = *af*(*ϕ*) + *bf*(*ψ*).

**Definition 6**

Denote by (*ϕ*_*n*_) a sequence of test functions and Φ another test function. We say that *ϕ*_*n*_ → Φ if the following holds:

1. there is an interval [*a*, *b*] that contains supp (Φ) and supp (*ϕ*_*n*_) for all *n*,
2. lim _*n*→*∞*_ *ϕ*_*n*_^(*k*)^(*t*) → Φ^(*k*)^(*t*) uniformly for *t* ∈ [*a*, *b*].

**Definition 7**

A functional *f* on **D** is continuous if it maps every convergent sequence in **D** into a convergent sequence in **C**. A continuous linear functional *f* on **D** is termed a generalized function. It is often called a distribution in the sense of Schwartz.

**Definition 8**

A function *f* : **R** → **C** is locally integrable if ∫_*a*_^*b*^ *f*(*t*)*dt* < *∞* for all *a*, *b*.

**Lemma 10**

Any continuous, including piecewise continuous, function is locally integrable.

**Lemma 11 (regular)**

Any locally integrable function *f* is a generalized function defined by

(42) $$\langle f,\phi \rangle =\overset{\infty }{\underset{-\infty }{\int }}f\left(t\right)\phi \left(t\right)dt<\infty .$$

In this case, *f* is called regular.

**Lemma 12**

Any generalized function has derivatives of all orders.

**Lemma 13**

There exists the Fourier transform of any generalized function.

**Definition 9 (rapid function)**

A function of rapid decay is a smooth function *ϕ* : **R** → **C** such that *t* ^*n*^ *ϕ* ^(*r*)^(*t*) → 0 as *t* → ±  *∞* for all *n*, *r* ≥ 0, where **C** is the space of complex numbers. The set of all functions of rapid decay is denoted by **S**.

**Lemma 14**

Every function belonging to **S** is absolutely integrable.

### 4.2. Proof of Existence

Define the norm and inner product of *X* ∈ *𝕊* by

(43) $${||X||}^{2}=\langle X,X\rangle =\overset{\infty }{\underset{0}{\int }}X^{2}\left(u\right)w\left(u\right)du,$$

where *w* ∈ **S**. Combining any *X* ∈ *𝕊* with its limit yields a Hilbert space that we denote again by *𝕊* without confusions.

Let *g* ∈ *𝕊* be a system function such that it transforms its input *X* ∈ *𝕊* to the output by

(44) y=(X⊗g)∈𝕊.

Denote the system by the operator *L*. Then, we purposely force the functionality of *L* such that it maps an element *X* ∈ *𝕊* to another element (*X* ⊗ *g*) ∈ *𝕊*. Note that *L* is a linear operator. In fact, according to Lemma 8, we have

(45) L(X∧g)=L(X)∧L(g).

In addition, from Lemma 9, we have

(46) L(X+K)=L(X)+K.

Therefore, *L* is a linear mapping from *𝕊* to *𝕊*.

Denote by **L** the space consisting of all such operators by

(47) L(𝕊,𝕊)=L(𝕊).

Then, from Lemmas 8 and 9, one can easily see that **L**(*𝕊*) is a linear space.

**Lemma 15 (archimedes criterion)**

For any positive real numbers *a* > 0 and *b* > 0, there exists positive integer *n* ∈ **Z** such that *na* > *b* (see [130]).

**Lemma 16 (archimedes)**

If *b* ∈ **R**, there exists *n* ∈ **Z** such that *b* < *n* (see [106]).

**Lemma 17**

An operator *T* : *X* ↦ *Y* is invertible if and only if there exists constant *m* > 0 such that for all *x* ∈ *X*, ||*Tx*|| ≥ *m*||*x*||, where *X* and *Y* are linear normed spaces (see [131]).

From the above discussions, we obtain the following theorem.

**Theorem 2 (existence)**

For *X*, *g* ∈ *𝕊* and *X*(0) ≠ 0 and *g*(0) ≠ 0, if *L*(*X*) = *X* ⊗ *g* or *L*_1_(*g*) = *g* ⊗ *X*, then both *L* and *L*_1_ are invertible. Consequently, the identity in the min-plus algebra exists.

**Proof**

Consider

(48) $$\begin{array}{cc} ||LX|| & =\sqrt{||X⊗g||}\\ & =\sqrt{\overset{\infty }{\underset{0}{\int }}{\left[\underset{0\leq u\leq t}{\inf\;}\left\{X\left(u\right)+g\left(t-u\right)\right\}\right]}^{2}w\left(u\right)du.} \end{array}$$

Since

(49) $$\underset{0\leq u\leq t}{\inf\;}\left\{X\left(u\right)+g\left(t-u\right)\right\}\geq \inf\;\left\{X\left(u\right)\right\}=X\left(0\right)$$

and *X*(*u*) ∈ *𝕊*, we have

(50) 0<X(0)≤X(u).

According to Lemmas 15 and 16, there exists *m* > 0 such that

(51) $$X\left(0\right)\geq m^{2}X\left(u\right).$$

Therefore,

(52) $$\begin{array}{cc} ||LX|| & \geq \sqrt{\overset{\infty }{\underset{0}{\int }}{\left[\inf\;\left\{X\left(u\right)\right\}\right]}^{2}w\left(u\right)du}\\ & =\sqrt{\overset{\infty }{\underset{0}{\int }}{\left[X(0)\right]}^{2}w\left(u\right)du}\\ & \geq m\sqrt{\overset{\infty }{\underset{0}{\int }}X{\left(u\right)}^{2}w\left(u\right)du}=m||X||. \end{array}$$

Similarly, if *L*_1_ ∈ **L**(*𝕊*)  is such that *L*_1_(*g*) = *g* ⊗ *X*, we have ||*L*_1_ *g*|| ≥ *m*_1_||*g*|| since *g*(0) ≠ 0, where *m*_1_ > 0 is a constant. Thus, according to Lemma 17, Theorem 2 holds.

**Note 7**

In Theorem 2, we need the conditions of *X*(0) ≠ 0 and *g*(0) ≠ 0. Since *X*(*t*) and *g*(*t*) are wide sense increasing, we need in fact *X*(0) > 0 and *g*(0) > 0.

## 5. Representation of Identity in Min-Plus Algebra

Express the Dirac-*δ* function by

(53) $$\delta \left(t\right)=\frac{1}{2\pi }+\frac{1}{\pi }\overset{\infty }{\underset{k=-\infty }{\sum }}\cos\;\left(kt\right).$$

For the purpose of distinguishing the identity we present from the conventional one, we denote *I*(*t*) as the identity in what follows instead of *I*_1_(*t*) as used in Section 3.

**Theorem 3 (representation)**

The identity in the min-plus algebra is expressed by

(54) $$I\left(t\right)=\underset{T\to 0}{\lim\;}\left[\frac{2}{T}+\frac{4}{T}\overset{\infty }{\underset{n=1}{\sum }}\cos\;\left(\frac{2n\pi t}{T}\right)\right].$$

**Proof**

Take the following into account

(55) $$\overset{\infty }{\underset{n=0}{\sum }}\delta \left(t-nT\right)\;\left(T>0\right).$$

Then, the identity in the discrete case is given by

(56) $$I\left(k\right)\;=\overset{\infty }{\underset{n=0}{\sum }}\delta \left(k-nT\right).$$

The identity in the continuous case is taken as the limit expressed by

(57) $$I\left(t\right)=\underset{T\to 0}{\lim\;}\overset{\infty }{\underset{n=0}{\sum }}\delta \left(t-nT\right).$$

Considering the Poisson's summation formula, we have

(58) $$I\left(k\right)\;=\frac{2}{T}+\frac{4}{T}\overset{\infty }{\underset{n=1}{\sum }}\cos\;\left(\frac{2n\pi k}{T}\right).$$

In the limit case,

(59) $$I\left(t\right)=\underset{T\to 0}{\lim\;}\left[\frac{2}{T}+\frac{4}{T}\overset{\infty }{\underset{n=1}{\sum }}\cos\;\left(\frac{2n\pi t}{T}\right)\right].$$

This completes the proof.

**Remark 8**

If one uses the representation in Theorem 3, the contradictions given in (38) and (41) vanish.

**Note 8**

The identity expressed by (59) is an asymptotic one.

## 6. Discussions

**Figure 3:**
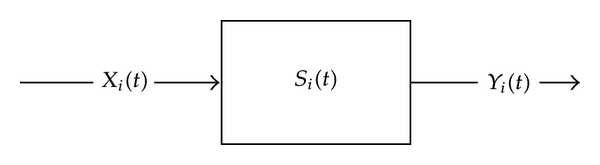
Single node with arrival and departure traffic.

We mention an application of min-plus algebra to CPNS. Denote by *Y*_*i*_(*t*) the accumulated function characterizing the output of the *i*th node (Figure 3). Then, the min-plus convolution can be used to establish the relationship between *X*_*i*_(*t*), *S*_*i*_(*t*), and *Y*_*i*_(*t*) by

(60) $$Y_{i}\left(t\right)\geq X_{i}\left(t\right)⊗S_{i}\left(t\right)=\underset{0\leq u\leq t}{\inf\;}\;\left\{S_{i}\left(u\right)+X_{i}\left(t-u\right)\right\}.$$

**Figure 4:**
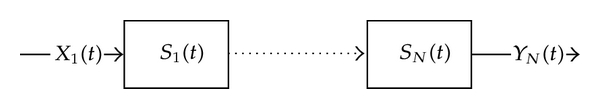
*N* tandem nodes with arrival and departure traffic.

Suppose a traffic function passes through *N* tandem nodes from the first node with the service curve *S*_1_(*t*) to the *N*th node with the service curve *S*_*N*_(*t*) to reach the destination as indicated in Figure 4. Denote the departure traffic of the *N*th node by *Y*_*N*_(*t*). Then,

(61) $$Y_{N}\left(t\right)\geq X_{1}\left(t\right)⊗S_{N}^{1}\left(t\right)=\underset{0\leq u\leq t}{\inf\;}\;\left\{S_{N}^{1}\left(u\right)+X_{1}\left(t-u\right)\right\},\;$$

where (see [132])

(62) $${\text{S}}_{N}^{1}\left(t\right)=S_{1}\left(t\right)⊗S_{2}\left(t\right)⊗\cdots ⊗S_{i}\left(t\right)\cdots ⊗S_{N}\left(t\right).$$

**Note 9**

Min-plus algebra can be used to linearize a nonlinear system as can be seen from (62). Thus, it may yet be used as a theory in the aspect of data transmission systems in CPNS.

## 7. Conclusions

We have proposed the problem regarding the conventional identity in the min-plus algebra. In addition, we have presented the proof that the identity in the min-plus algebra exists in the domain of generalized function. Moreover, we have given the asymptotic expression of the identity in the system of min-plus algebra.
